# Supplementary material for: FERN – a Java framework for stochastic simulation and evaluation of reaction networks
Source: BMC Bioinformatics. 2008 Aug 29;9:356. doi: 10.1186/1471-2105-9-356 (PMC2553347; doi:10.1186/1471-2105-9-356)
Supplement: Additional file 1 — FERN distribution, Version 1.3. This archive contains the FERN source code and binaries as well as documentation and example models in FernML and SBML. [file 1471-2105-9-356-S1.zip › fern/doc/javadoc/index-files/index-15.html]

P-Index


---


|  |  |  |
| --- | --- | --- |
| **Overview** \| Package \| Class \| Use \| **Tree** \| **Deprecated** \| **Index** \| **Help** | |  |
| **PREV LETTER**   **NEXT LETTER** | **FRAMES**    **NO FRAMES**     **All Classes** |  |


A B C D E F G H I J L M N O P R S T U V 

---


## **P**

**paint(Graphics)** - Method in class fern.tools.gnuplot.GnuPlot: **performReaction(int, int)** - Method in class fern.network.AmountManager: Reflects a (multiple) firing of a reaction by adjusting the populations of the reactants and the products. **performStep(SimulationController)** - Method in class fern.simulation.algorithm.AbstractBaseTauLeaping: Performs a tau leaping step. **performStep(SimulationController)** - Method in class fern.simulation.algorithm.GibsonBruckSimulator: **performStep(SimulationController)** - Method in class fern.simulation.algorithm.GillespieEnhanced: **performStep(SimulationController)** - Method in class fern.simulation.algorithm.GillespieSimple: **performStep(SimulationController)** - Method in class fern.simulation.algorithm.HybridMaximalTimeStep: **performStep(SimulationController)** - Method in class fern.simulation.Simulator: Performs one simulation step. **plot()** - Method in class fern.tools.gnuplot.GnuPlot: Calls gnuplot to plot the data. **present()** - Method in class fern.benchmark.SimulatorCorrectness: Gets called after some iterations of the method `benchmark` in the base class. **present()** - Method in class fern.benchmark.SimulatorFireTypes: Present results of this benchmark is gnuplot and text to stdout. **present()** - Method in class fern.benchmark.SimulatorPerformance: Is called after `getShowSteps` iterations. **present()** - Method in class fern.benchmark.SimulatorRandomNumbers: Present results of this benchmark is gnuplot and text to stdout. **present()** - Method in class fern.benchmark.SimulatorTime: Present results of this benchmark is gnuplot and text to stdout. **print()** - Method in class fern.simulation.observer.Observer: Gets called after the `finish` call and prints the results of this observer if a `PrintWriter` is registered. **Probability** - Interface in fern.tools.functions: Function delegate for conditional probabilities. **Probability.Constant** - Class in fern.tools.functions: Implementation that yields a constant (and hence independent) probability. **Probability.Constant(double)** - Constructor for class fern.tools.functions.Probability.Constant: **Probability.ReactionProbability** - Class in fern.tools.functions: Implementation for the reaction probability for the autocatalytic network creation. **Probability.ReactionProbability(double, int)** - Constructor for class fern.tools.functions.Probability.ReactionProbability: **productHistos** - Variable in class fern.simulation.algorithm.AbstractBaseTauLeaping: **propensitiyCalculator** - Variable in class fern.network.AbstractNetworkImpl: Stores the `PropensityCalculator` of the network. **PropensityCalculator** - Interface in fern.network: A `PropensityCalculator` is the way, a `Network` tells a `Simulator` how to calculate propensities for the reactions.

---


|  |  |  |
| --- | --- | --- |
| **Overview** \| Package \| Class \| Use \| **Tree** \| **Deprecated** \| **Index** \| **Help** | |  |
| **PREV LETTER**   **NEXT LETTER** | **FRAMES**    **NO FRAMES**     **All Classes** |  |


A B C D E F G H I J L M N O P R S T U V 

---
